# The influence of N and S poles of static magnetic field (SMF) on *Candida albicans* hyphal formation and antifungal activity of amphotericin B

**DOI:** 10.1007/s12223-019-00686-3

**Published:** 2019-02-20

**Authors:** Dariusz Sztafrowski, Jakub Suchodolski, Jakub Muraszko, Karel Sigler, Anna Krasowska

**Affiliations:** 1 Faculty of Electrical Engineering, Wrocław University of Science and Technology, ul. Wybrzeże Wyspiańskiego 27, 50-370 Wrocław, Poland; 2 Department of Biotransformation, Faculty of Biotechnology, University of Wroclaw, ul. F. Joliot-Curie 14a, 50-383 Wrocław, Poland; 3 Institute of Microbiology of the Czech Academy of Sciences, Vídeňská 1083, 142 20 Prague, Czech Republic

## Abstract

Due to the increasing number of *Candida albicans*’ infections and the resistance of this pathogenic fungus to drugs, new therapeutic strategies are sought. One of such strategies may be the use of static magnetic field (SMF). *C. albicans* cultures were subjected to static magnetic field of the induction 0.5 T in the presence of fluconazole and amphotericin B. We identified a reduction of *C. albicans* hyphal length. Also, a statistically significant additional effect on the viability of *C. albicans* was revealed when SMF was combined with the antimycotic drug amphotericin B. The synergistic effect of this antimycotic and SMF may be due to the fact that amphotericin B binds to ergosterol in plasma membrane and SMF similarly to MF could influence domain orientation in plasma membrane (PM).

## Introduction

*Candida albicans* is a microorganism forming part of human microflora, which under immunosuppression causes opportunistic infections (Dadar et al. 2018). Chronic mucocutaneous candidiasis (CMC) is characterized by infections of the skin, nails, and oral and genital mucosae (Puel et al. 2011). However, under high immunodeficiency of the host, *C. albicans* enters the bloodstream and induces systemic infections with a mortality rate ranging from 30 to 80% (Gunsalus and Kumamoto 2016; Whaley et al. 2017). After the transition from yeast to hyphal form, *C. albicans* penetrates the host’s physiological barriers (Richardson et al. 2018). *C. albicans* infections are characterized by increasing resistance to traditional antifungal agents, such as fluconazole and amphotericin B (Pfaller 1996; Mah and O’Toole 2001). The mechanisms of resistance include overproduction of membrane drug efflux transporters (mainly Cdr1p belonging to ATP-binding cassette (ABC) family) (Hernáez et al. 1998), or changes in the expression of genes involved in ergosterol biosynthesis (mainly *ERG11* gene encoding lanosterol 14α-demethylase) (Martel et al. 2010). The increasing resistance of *C. albicans* to drugs is associated with the need to develop new treatment strategies; one of them may be the use of SFM.

Living organisms are permanently exposed to constant Earth’s magnetic field (MF) (Zhang et al. 2018; Cao and Pan 2018). The number of MF applications in medical therapies has been increasing over the last decades and now includes magnetotherapy, magnetic stimulation (MS), and transcranial magnetic stimulation (TMS) (Sztafrowski et al. 2018).

Biological processes are currently being monitored under the influence of static magnetic field (SMF) and alternating MF, the value of which is several orders larger than the Earth’s MF (Sztafrowski et al. 2017). In vitro, SMF exposure can reduce the number of viable cells in melanoma, ovarian carcinoma, and lymphoma cell lines (Raylman et al. 1996). In clinical trials, SMF induces analgesic benefits in patients with: symptomatic diabetic peripheral neuropathy (DPN) (Weintraub et al. 2003), fibromyalgia (Alfano et al. 2004), rheumatoid arthritis (RA) (Segal et al. 2001), and postpolio (Vallbona et al. 1997).

Unlike a large number of publications about the influence of SMF on human cells, information about its effect and mechanism of toxicity on microorganisms is less known. SMF has no significant effect on the growth of pathogenic microorganisms such as *Escherichia coli* or *Staphylococcus aureus* (Grosman et al. 1992) but it induces antibiotic resistance in *E. coli* (Stansell et al. 2001). In phytopathogenic fungi, SMF was shown either to stimulate (*Alternaria alternata* and *Coelophora inaequalis*) or reduce conidia development (*Fusarium oxysporum* and *Fusarium culmorum*) (Albertini et al. 2003; Nagy and Fischl 2004).

Since there are limited data on the influence of SMF on microorganisms, especially on yeast and pathogenic yeast-like fungi, the aim of this study was to check whether SMF has an impact on general viability of *C. albicans* hyphal transition and its susceptibility to fluconazole and amphotericin B.

## Materials and methods

### Chemicals, strains, and growth conditions

Chemicals and reagents used in this study were purchased from the following sources: fluconazole and conventional amphotericin B (Sigma-Aldrich; Poznań, Poland); d-glucose and bacteriological agar (Lab Empire; Rzeszów, Poland); peptone and yeast extract (YE) (Diag-med; Warszawa, Poland); and fetal bovine serum (FBS) (Thermo Fisher; Warszawa, Poland).

*C. albicans* strain CAF2-1 (genotype: *ura3Δ::imm434/URA3*) was a kind gift of Prof. D. Sanglard (Lausanne, Switzerland) (Fonzi and Irwin 1993). It was routinely grown at 28 °C on YPD medium (2% glucose, 1% peptone, 1% YE) with agitation (120 rpm). Agar in a final concentration of 2% was used for medium solidification.

### Exposure of biological material to SMF

**Fig. 1:**
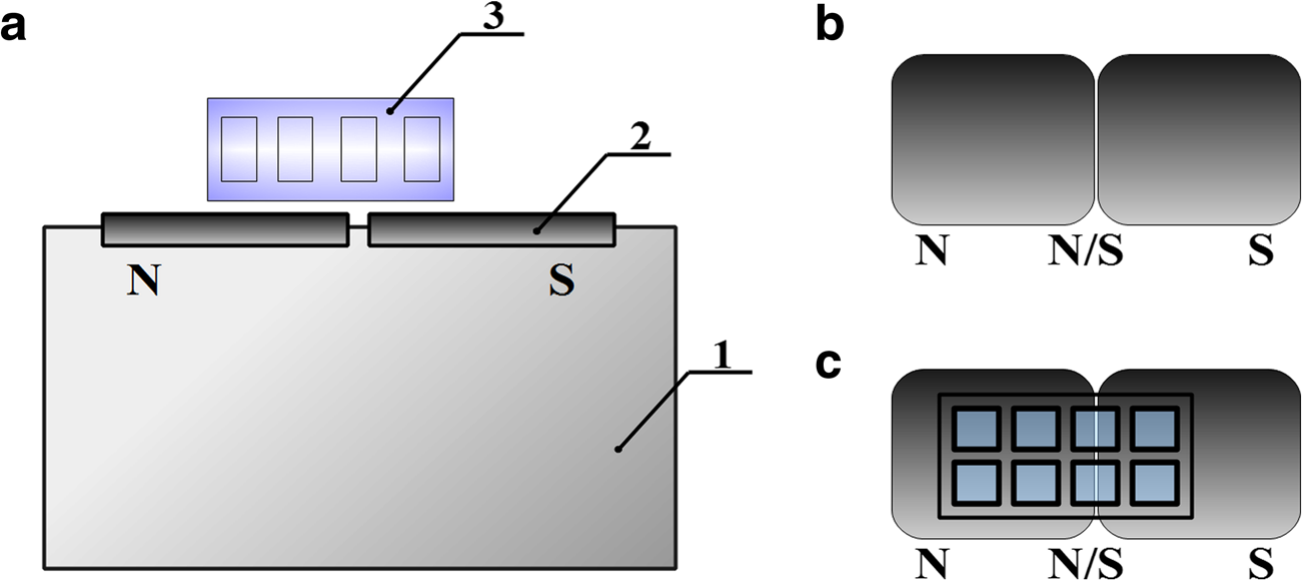
Schematic representation of the testing stand for exposure of *C. albicans* cells to SMF (**A**, side view); the base 1 housed the two neodymium magnets 2, on which 8-well culture chambers 3 were located. Neodymium magnet (**B**, view from above) forms the N pole and its counterpart the S pole. Location of 8-well culture chambers on the neodymium magnet (**C**) allowed for simultaneous exposure of 2 biological replicates to each (N, S, N/S) pole

The schematic representation of the testing stand is given in Fig. 1. Two permanent magnets were used as a source of magnetic field. The source of the magnetic field is a neodymium magnet which is made of N48 with the following dimensions: length, 60 mm; width, 60 mm; height, 25 mm; and the magnetization direction, along the dimension of 25 mm. The mass of the magnetic element is 674 g. Magnetic properties of the source of the magnetic field are as follows: the remanence Br 1.38–1.42 T (Tesla, abbr. T – SI-derived unit of magnetic induction; 1 T is interpreted as a value of magnetic induction which, for a charge of 1 C, moving at a speed of 1 m/s perpendicular to the magnetic field line, acts with a Lorentz force of 1 N), normal coercivity HcB min 835 kA/m, intrinsic coercivity HcJ min 875 kA/m, and magnetic energy density (BH) max 366–390 kJ/m^3^. The direction of magnetization along the height means that the surface of magnetic element that is perpendicular to the height forms the “N” pole and its counterpart on the opposite end of the magnet forms the “S” pole. Magnetic field induction close to the edge of the surface of the magnetic pole (maximum) with a distance of 0.7 mm is 0.5 T. Eight-well culture chambers were placed on the top of the neodymium magnet (Fig. 1B) so that 4 wells were simultaneously exposed to N pole, 2 to N/S pole, and 2 to S pole (Fig. 1C). To maintain identical growth conditions, only 2 out of 4 wells exposed to N pole were inoculated each time. Control experiments were performed using 8-well culture chambers not exposed to SMF.

### The impact of SMF on general *C. albicans* viability

Twenty-four-hour cultures of *C. albicans* (YPD medium; 120 rpm; 28 °C) were centrifuged (5 min, 4.5 k rpm), washed with fresh YPD medium, and resuspended in YPD medium of A_600_ = 0.1 (corresponding to cell concentration of 1.4 × 10^6^ cfu/mL). Eight-well culture chambers were inoculated as described in “Exposure of biological material to SMF” section, to a final volume of 300 μL and cultured for 24 h at 28 °C. The material was then transferred to a 96-well plate and A_600_ was measured using ASYS UVM 340 (Biogenet) microplate reader.

### The impact of SMF on yeast to yeast-to-hyphae transition

Twenty-four-hour cultures of *C. albicans* (YPD medium; 120 rpm; 28 °C) were centrifuged (5 min, 4.5 k rpm), washed with fresh YPD medium, and resuspended in YPD medium of A_600_ = 0.4 (corresponding to a cell concentration of 5.9 × 10^6^ cfu/mL). At this point, control microscopic preparation was made (negative control). The exposure to SMF was performed in 8-well culture chambers, as described in “Exposure of biological material to SMF” section (positive control: induction of hyphal transition with no exposure to SMF). To induce hyphal transition, the suspensions were treated with FBS (final conc. = 10%) for 2 h at 37 °C. The samples were observed under Zeiss Axio Imager A2 microscope equipped with Zeiss Axiocam 503 mono microscope camera for the assessment of cell morphology (*n* = 50–100 cells in four repetitions). The length (μm) of straight hyphae was measured using Zeiss ZEN 2 Blue software.

### The impact of SMF on drug susceptibility of *C. albicans*

Twenty-four-hour culture of *C. albicans* (YPD medium; 120 rpm; 28 °C) was centrifuged (5 min, 4.5 k rpm), washed with fresh YPD medium, and resuspended in YPD medium of A_600_ = 0.1 (corresponding to cell concentration of 1.4 × 10^6^ cfu/mL). Eight-well culture chambers were inoculated, as described in “Exposure of biological material to SMF” section to a final volume of 300 μL. Each well was treated with fluconazole (final conc. = 2 or 4 μg/mL) or amphotericin B (final conc. = 0.063 or 0.125 μg/mL) and cultured for 24 h at 28 °C. Such concentrations of antibiotics have been selected that lower the A_600_, but do not kill the cells. Thereafter, the material was transferred to a 96-well plate and A_600_ was measured using ASYS UVM 340 (Biogenet) microplate reader.

### Statistical analysis

Each experiment was performed at least in triplicate. Statistical significance was determined using the Tukey-Kramer HSD post hoc test after the one-way ANOVA (*α* = 0.05).

## Results

In each experiment, *C. albicans* CAF2-1 cells were divided into four groups. Control cells were not subjected to the influence of SMF. Other cell groups were subjected to different conditions in the SMF magnet zones: at the north pole (N), at the south pole (S), or between the north and south poles (N/S) (Fig. 1). Most of the data were presented in a twofold manner for comprehensive interpretation: box-and-whiskers plot (minimal and maximal data, median, first and third quartiles (Q_1_; Q_3_)) and histograms (average ± standard deviation (SD)).

**Fig. 2:**
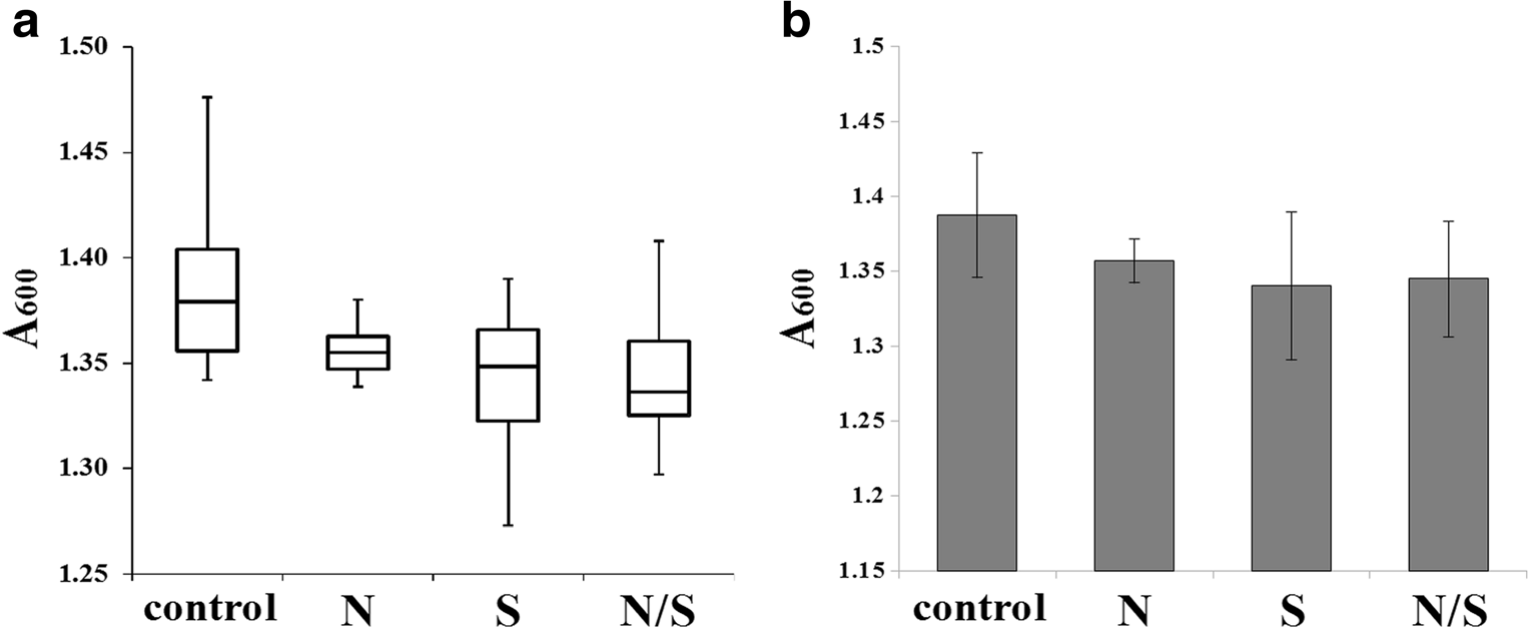
The viability (A_600_ after 24-h incubation at 28 °C) of *C. albicans*’ cells exposed to SMF (N, S, N/S poles) in comparison with cells not exposed to SMF (control). Data are presented as box-and-whiskers plot (**A**), which includes minimal and maximal A_600_, median, and Q_1_ and Q_3_. and histogram (**B**), which includes average ± SD. Statistical analysis was performed by one-way ANOVA

Figure 2 shows the viability of *C. albicans* CAF2-1 cells after a 24-h exposure to SMF at 28 °C. Median A_600_ of cells exposed to all SMF zones (N, S, N/S) was lower than the control (Fig. 1A), the lowest A_600_ being in the case of exposure to the S/N pole. Maximal A_600_ was at least 7.2% lower in cells exposed to SMF; minimal A_600_ was 5% and 3.25% lower in case of exposure to the S and N/S poles, respectively. Additionally, Q3 data of cells exposed to SMF are considerably lower than those of Q1 of the control. The average viability (Fig. 2B) was reduced only by 2.2% (N pole) to 3.4% (S pole). However, the acquired data were not significant (*p* > 0.05).

**Fig. 3:**
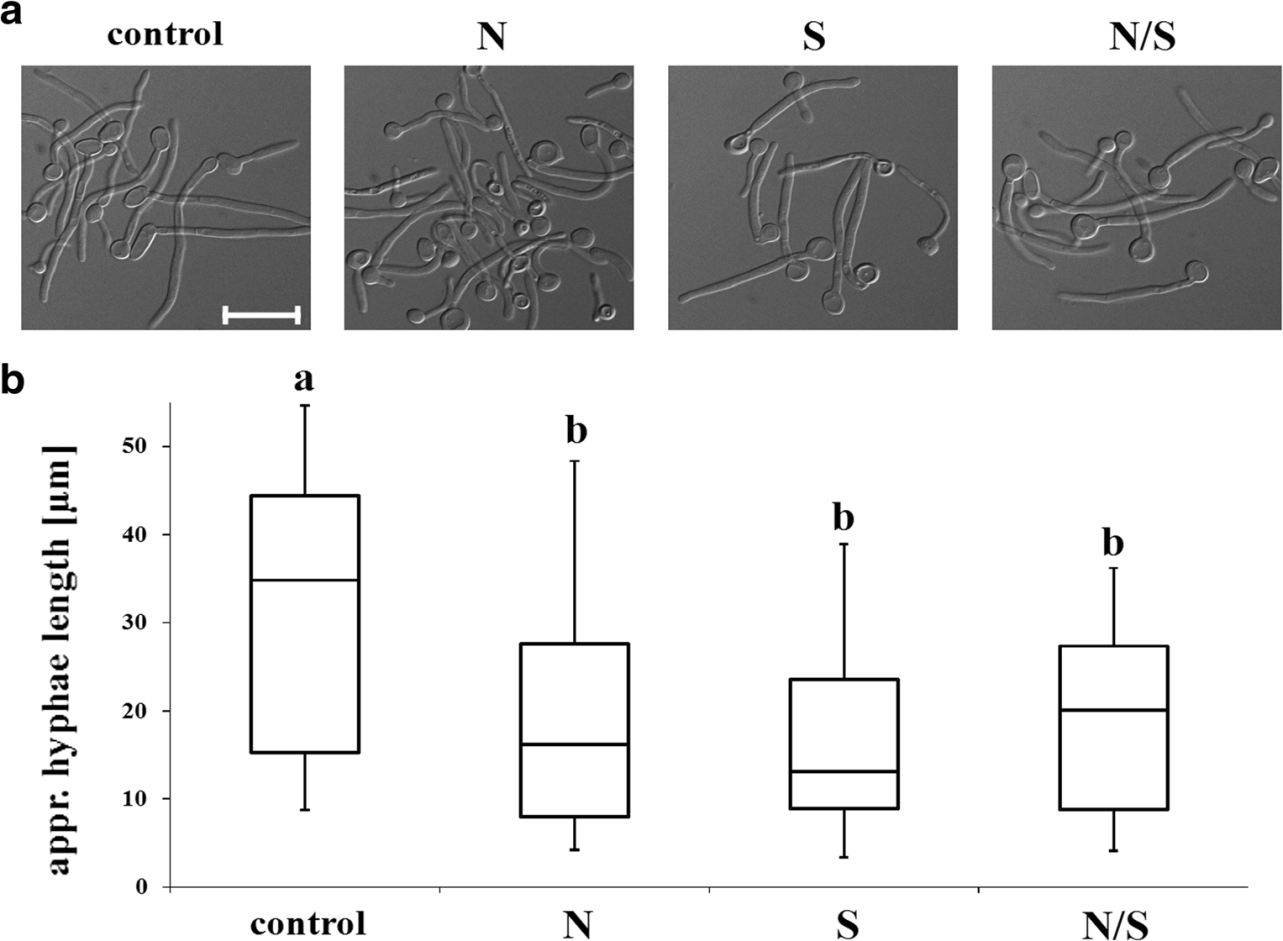
Morphology of *C. albicans* hyphae after 2-h induction with FBS at 37 °C exposed to SMF (N, S, or N/S poles) in comparison with untreated cells (control) (**A**, scale bar = 20 μm). Hyphal length: minimal and maximal, median, Q_1_ and Q_3_. Statistical analysis was performed by ANOVA – Tukey-Kramer’s test; included on box-and-whiskers plot (**B**). Lowercase letter “a” indicates the difference from the N, S, or N/S pole when *p* < 0.05; lowercase letter “b” indicates the difference from the control when *p* < 0.05

*C. albicans* forms hyphae after induction with FBS at 37 °C (conditions which mimic the environment of the infected host niche). Exposure towards SMF does not inhibit this process (Fig. 3A) and in this case, all cells formed hyphae. The percentage of filaments was as follows: control, 94.8 ± 1.1; N pole, 94.1 ± 2.3; S pole, 92 ± 2.2; and N/S pole, 98.5 ± 3.1. As a control, the cell morphology was checked before hyphal induction and only blastospores without visible hyphae or germ tubes were observed (data not shown). However, a population of shorter hyphae (germ tubes) was observed after exposure to SMF (Fig. 3B). The median length of hyphae decreased from 34.8 μm (control) to 16.2, 13.1 and 20.1 μm after exposure to N, S, and N/S poles, respectively. The length in control sample was between 8.7 (minimum) and 54.7 (maximum) μm. The minimal length after exposure to SMF was 4.2 (N pole), 3.4 (S pole), and 4.1 (N/S pole) μm, whereas the maximal length increased to 48.3 (N pole), 38.9 (S pole), and 36.2 (N/S pole) μm. Q_3_ of hyphal length in cells exposed to SMF was considerably lower, with a value much below the average length of control hyphae. The average length of hyphae formed by cells not exposed to SMF was 32.9 ± 15.1 μm, whereas exposure to N, S, and N/S resulted in 18.2 ± 10.8, 16 ± 9.3 and 19.2 ± 10.5 μm average hyphal length, respectively. Statistical analysis yielded considerably low *p* value (9.11E – 10).

**Fig. 4:**
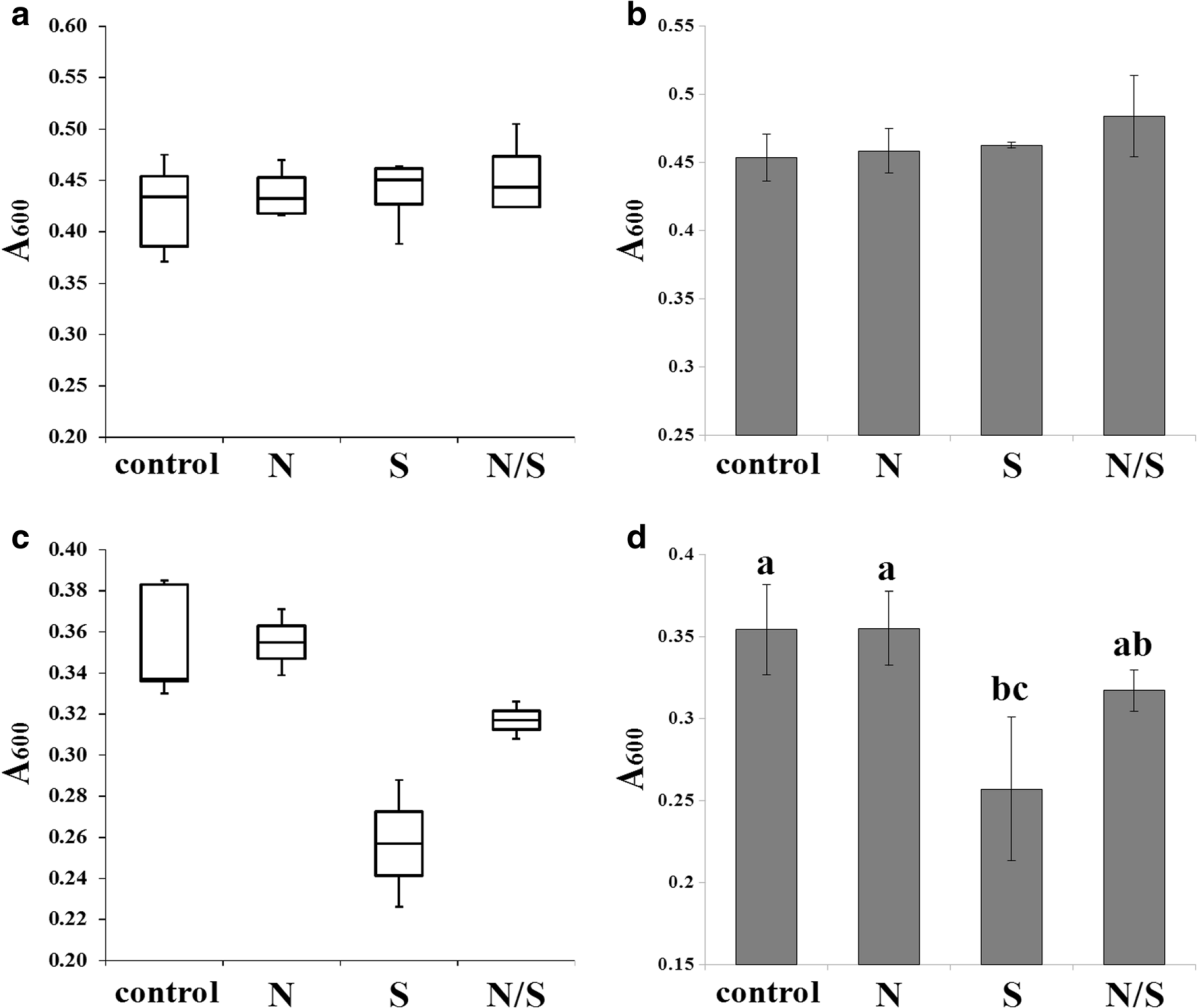
Fluconazole (2 μg/mL A + B; 4 μg/mL C + D) susceptibility (A_600_ after 24-h incubation at 28 °C) of *C. albicans* CAF2-1 cells exposed to SMF (N, S, N/S poles) in comparison with cells not exposed to SMF (control). Data are presented as box-and-whiskers plots (**A**, **B**) include minimal and maximal A_600_, median, and Q_1_ and Q_3_. Histograms include average ± SD (**C**, **D**). Statistical analysis was performed by ANOVA – Tukey-Kramer’s test; included on the histogram (different lowercase letters in D indicate significant differences at *p* < 0.05)

**Fig. 5:**
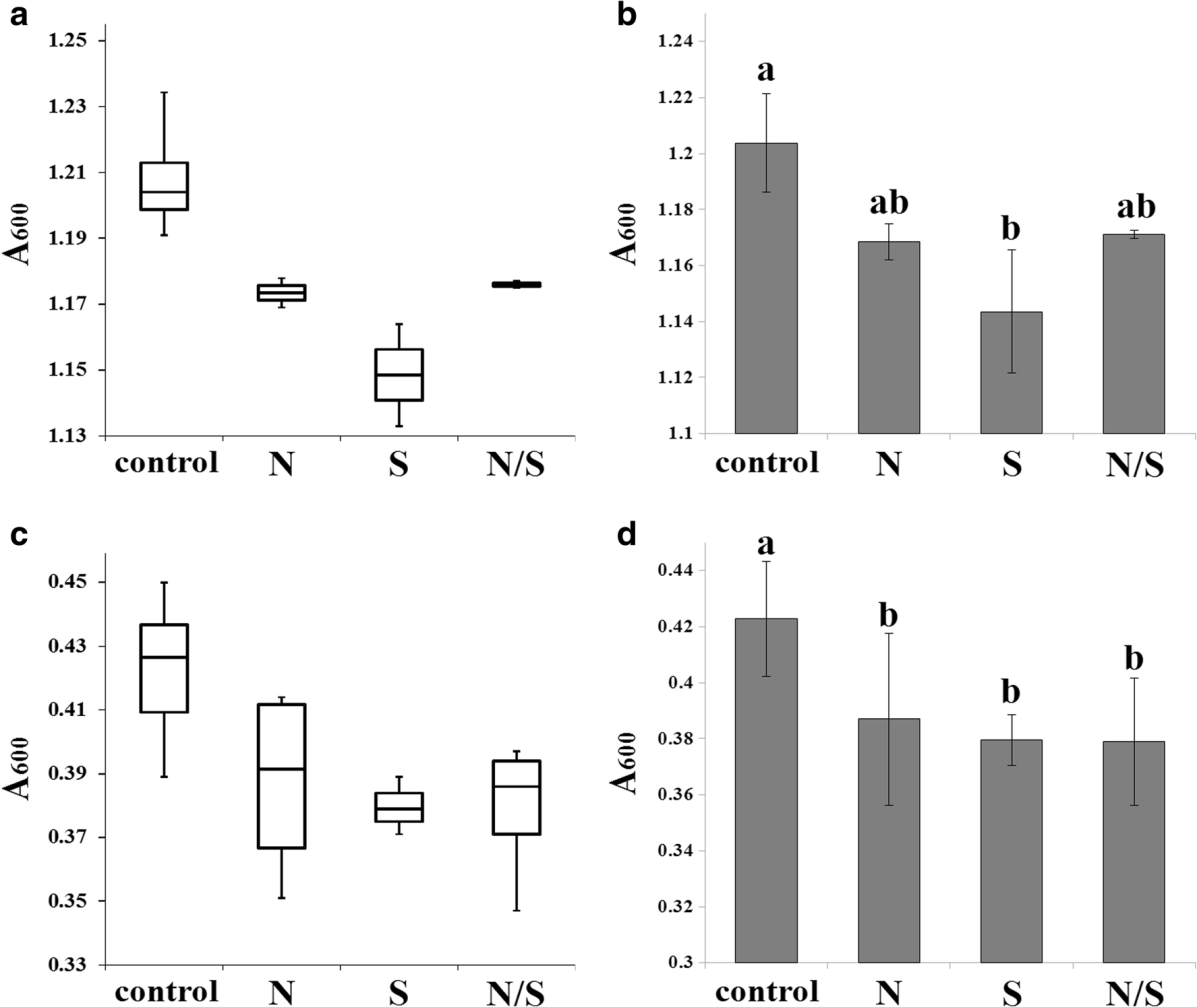
Amphotericin B (0.0625 μg/mL A + B; 0.125 μg/mL C + D) susceptibility (A_600_ after 24-h incubation at 28 °C) of *C. albicans* CAF2-1 cells exposed to SMF (N, S, N/S poles) in comparison with cells not exposed to SMF (control). Data are presented as box-and-whiskers plots (**A**, **B**) that include minimal and maximal A_600_; median; Q_1_ and Q_3_. Histograms (**C**, **D**) include average ± SD. Statistical analysis was performed by ANOVA – Tukey-Kramer’s test, included on histograms B + D. Lowercase letters indicate significant differences at *p* < 0.05

In the second part of the experiments, we examined the effect of SMF plus two antimycotics, fluconazole and amphotericin B, on *C. albicans* (Figs. 4 and 5).

Data obtained for various drug concentrations are shown in separate graphs for a clearer presentation.

*C. albicans* cells exposed to SMF in the presence of 2 μg/mL fluconazole displayed no significant changes in median, Q_1_ (Fig. 4A) and average (Fig. 4B) of A_600_. Unexposed cells display lower Q_3_ of A_600_, and cells exposed to S pole show lower minimal A_600_. Cells exposed to N/S pole display higher maximum A_600_. In the presence of 4 μg/mL fluconazole, the most noticeable is the higher susceptibility of *C. albicans* cells exposed to S pole, reflected as lower parameters of A_600_: minimum, maximum, median, and Q_1_ and Q_3_ (Fig. 4C), as well as the average (Fig. 4D). The result is significant at *p* = 0.021.

Treatment of cells with 0.0625 μg/mL amphotericin B in the presence of SMF resulted in lower median A_600_ (Fig. 5A), with values of 1.17 in the case of N and N/S poles and 1.14 in the case of S pole (1.2 in control). A_600_ of unexposed cells was between 1.19 (minimum) and 1.21 (maximum). The minimal A_600_ after exposure to SMF was 1.16 (N pole), 1.13 (S pole), and 1.17 (N/S pole), whereas the maximal A_600_ increased to 1.17 (N pole), 1.16 (S pole), and 1.17 (N/S pole). Q_3_ data of cells exposed to N, S, and N/S are considerably lower (1.17, 1.15, and 1.17, respectively) than Q_3_ and Q_1_ of A_600_ in unexposed cells (1.21 and 1.19, respectively). Average A_600_ of cells exposed to both N and N/S poles (both = 1.17) was lower than A_600_ of unexposed cells (= 1.2); the lowest average A_600_ (= 1.14) was obtained after exposing cells to the S pole, with statistical significance (*p* = 0.008).

A similar trend was observed after exposing *C. albicans* cells to SMF in the presence of 0.125 μg/mL amphotericin B (Fig. 5 C, D). A_600_ of unexposed cells was between 0.39 (minimum) and 0.45 (maximum), with a median at 0.43. The minimal A_600_ after exposure towards SMF was 0.35 (N pole), 0.37 (S pole), and 0.35 (N/S pole); the maximal A_600_ was 0.41 (N pole), 0.38 (S pole), and 0.39 (N/S pole) and the median A_600_ was with a value of 0.39 (N pole), 0.38 (S pole), and 0.39 (N/S pole). Q_3_ of A_600_ in cells exposed to SMF was in all cases lower than the median A_600_ of unexposed cells and, in the case of cells exposed to S and N/S poles, lower than Q_1_ of A_600_ in unexposed cells. The average A_600_ of cells exposed to both N, S, and N/S poles in the presence of 0.125 μg/mL amphotericin B (0.39, 0.38 and 0.38, respectively) was lower than A_600_ of unexposed cells (= 0.42). The results obtained were significant at *p* = 0.003.

## Discussion

Considering our preliminary results, it seems that the potential use of a SMF in antifungal therapy could be a new option of supporting treatment for *Candidas*’ infections. Previously, an inhibitory effect of SMF on cancer cell lines was identified (Raylman et al. 1996; Sabo et al. 2002; Luo et al. 2016; Sztafrowski et al. 2018) with no influence on prokaryotic bacterial spp. (Grosman et al. 1992). This led us to the conclusion that SMF may inhibit eukaryotic fungal cells. The rate of *C. albicans* growth inhibition is rather slight (7.2–8.6% reduction in maximal A_600,_ Fig. 2A). The SMF inhibitory effect towards phytopathogenic fungi was at a similar rate (5–10%) (Nagy and Fischl 2004). However, the response of fungi to SMF appears to depend on the species, because, e.g., SMF inhibited the growth of *Aspergillus niger* (Mateescu et al. 2011).

We identified a significant reduction of *C. albicans*’ hyphal length (Fig. 3). SMF also inhibited myceliar growth of phytopathogenic *F. culmorum* (Albertini et al. 2003) and pathogenic necrotroph *Syspastospora parasitica* (Mazurkiewicz-Zapalowicz et al. 2015)*.* This activity does not seem to be universal, since SMF had no impact on myceliar growth in *Tuber borchii* fungus (Potenza et al. 2012). In the case of *C. albicans*, SMF does not completely inhibit hyphal formation, but it should be taken into consideration that the median hypha length was from 34.8 μm (control, Fig. 3B) to 16.2 (N pole), 13.1 (S pole), and 20.1 (N/S pole), i.e., respectively 53, 62, and 42% length reduction. The ability of *C. albicans* to form hyphae at 37 °C is one of the virulence determinants and is connected with biofilm formation and further colonization of tissues (Suchodolski et al. 2017). Moreover, *C. albicans* deprived of the ability to form hyphae becomes avirulent in mouse models (Diez-Orejas et al. 1997; Lo et al. 1997; Calera et al. 2000; Cao et al. 2006; Ku et al. 2017).

Sztafrowski et al. 2018 identified an additive effect of SMF on HL-60 cancer cell line treatment with busulfan cytostatic. In the case of candidiasis treatment, a combination of azole/polyenes with other drugs/treatment strategies is highly desirable (Fiori and Van Dijck 2012; Perlin 2015). The combination of fluconazole with SMF resulted in visible growth inhibition only with 4 μg/mL concentration and S pole (Fig. 4). The result was significant according to ANOVA – Tukey-Kramer’s test; however, it was not observed when the fluconazole concentration was increased (data not shown). On the other hand, a statistically significant additive effect can be seen when SMF was combined with amphotericin B (Fig. 5). Amphotericin B binds to ergosterol in the plasma membrane (PM) with subsequent PM permeabilization and lethal effect (Gray et al. 2012). MF was shown to influence domain orientation in PM (Beck et al. 2010). Ruzic et al. 1997 found that sinusoidal MF leads to an increase of ergosterol content in mycorrhizal fungus *Pisolithus tinctorius.* It is known that *C. albicans* hyphal formation depends on sphingolipid-ergosterol domains (Pasrija et al. 2005a, b; McCourt et al. 2016; Wu et al. 2018), so it is possible that SMF influences plasma membrane organization.

In all experiments, the S pole generated the most promising results: lowest minimal and average A_600_ of *C. albicans* (Fig. 2); hyphal length reduction, the lowest minimal length, median, and average (Fig. 3B); a possible combination with fluconazole (Fig. 4); and the highest and most statistically significant additive effect with amphotericin B (Fig. 5). Our results suggest that SMF may have a potential in *C. albicans* treatment by influencing hypha formation and, especially, within amphotericin B treatment. However, this technique must be further studied and improved for future research and application.
